# When the Heart and Hip Collide: The Interplay Between Atrial Fibrillation and Neck of Femur Fractures

**DOI:** 10.3390/jcdd13070334

**Published:** 2026-07-16

**Authors:** Hannah Faherty, Thin Ei Hlaing, Khushi Thakkar, Mahir Hamad, Ahmed Hassan, Abdullah K. Ahmed, Musaab Ahmed, Mohamed T. Hassan, Mohamed H. Ahmed

**Affiliations:** 1 Department of General Surgery, Milton Keynes University Hospital NHS Foundation Trust, Milton Keynes MK6 5LD, UK; hannah.faherty@mkuh.nhs.uk; 2 Division of Acute Medicine, The James Cook University Hospital NHS Trust, Middlesbrough TS4 3BW, UK; drthineihlaing@gmail.com (T.E.H.); m.hamad@nhs.net (M.H.); 3 Department of Medicine, Milton Keynes University Hospital NHS Foundation Trust, Milton Keynes MK6 5LD, UK; khushi.thakkar@mkuh.nhs.uk; 4 Faculty of Medicine, Alexandria University, Alexandria 21500, Egypt; ahmed.mohamed2133@alexmed.edu.eg; 5 Tele-Geriatric Research Fellowship, Geriatric Division, Family Medicine Department, Michigan State University, East Lansing, MI 48824, USA; abdallahakhalid@gmail.com; 6 College of Medicine, Ajman University, Ajman P.O. Box 346, United Arab Emirates; m.omer@ajman.ac.ae; 7 Kasr Alainy Faculty of Medicine, Cairo University, Cairo 12613, Egypt; mohamed_tar_hassan@students.kasralainy.edu.eg; 8 Department of Medicine for Older People, North Cheshire and Mersey NHS Foundation Trust, Warrington WA5 1QG, UK; 9 Department of Diabetes, North Cheshire and Mersey NHS Foundation Trust, Warrington WA5 1QG, UK; 10 Faculty of Medicine and Health Sciences, University of Buckingham, Buckingham MK18 1EG, UK

**Keywords:** atrial fibrillation, fracture neck of femur and orthogeriatric

## Abstract

The association of atrial fibrillation (AF) and neck of femur fractures (NOF) are common in old people, creating a complex clinical scenario with significant implications for morbidity, mortality, and healthcare systems. This narrative review explores the bidirectional relationship between AF and NOF, focusing on shared risk factors, pathophysiological links, and challenges in clinical management, and also reviews the benefit of an orthogeriatric model. Advanced age, frailty, osteoporosis, polypharmacy, and cardiovascular comorbidities predispose patients to both conditions, while AF itself increases fall risk through haemodynamic instability, syncope, and adverse effects of rate- or rhythm-controlling medications. Importantly, the physiological stress of hip fracture and subsequent surgery can precipitate new-onset or worsening AF via inflammatory, neurohormonal, and metabolic mechanisms. The main challenge for ortho-geriatricians lies in anticoagulation management and preoperative and postoperative management. While anticoagulation reduces thromboembolic risk in AF, it increases perioperative bleeding risk in patients with NOF, often leading to delays in surgery that are independently associated with poorer outcomes. This review examines the current evidence regarding perioperative anticoagulation strategies, timing of surgery, and postoperative resumption of therapy. In addition, the review examines important outcome parameters such as mortality, stroke, bleeding, length of hospital stay, and functional recovery. This highlights the importance of not only improving multidisciplinary care involving orthopaedics, cardiology, geriatrics, and anaesthesia to optimise outcomes, but also enhancing risk stratification. Standardised perioperative pathways and integrated geriatric–cardiac assessment may help mitigate complications. Therefore, understanding how AF and NOF interact is key to delivering holistic, patient-centred care for an increasingly elderly population in orthogeriatric wards.

## 1. Introduction

Atrial fibrillation (AF) and neck of femur (NOF) fractures are both common in the elderly population, and their relationship is closely linked. Individuals with AF are more likely to fall, increasing the risk of NOF fractures. At the same time, patients who present with NOF fractures are often older and frailer, and many have underlying AF, whether or not it directly contributed to the fall. Age appears to be the key factor connecting these two conditions. The prevalence of AF rises significantly with age, affecting more than 10% of those over 80 years [1]. AF is the most common sustained cardiac arrhythmia globally [2]. In 2010, the prevalence of AF rose to 596.2 per 100,000 population in men (95% UI, 558.4–636.7) and 373.1 per 100,000 population in women (95% UI, 347.9–402.2), representing a substantial increase compared with figures from 1990 [1]. The recent literature highlights a continued increase; between 2010 and 2019, the global prevalence of AF has risen markedly from 33.5 million to 59 million individuals living with AF [3]. Globally, this rapidly expanding burden of AF is largely attributable to the increasing prevalence of chronic illnesses in the context of an ageing world population. The highest incidence and prevalence rates are reported in North America, Western Europe, East Asia, and Australia/New Zealand; however, data remains limited in Africa and South America. Although total morbidity and mortality continue to rise, age-adjusted annual rates have remained relatively stable, reflecting the demographic shift towards an older global population [2].

Similarly, hip fractures are also strongly age-associated, with more than 90% occurring in individuals over 65 years of age, most commonly as a result of falls and underlying osteoporosis [4]. They represent a serious clinical condition associated with a high risk of mortality, and, even amongst survivors, there are substantial reductions in quality of life, primarily due to impaired mobility and loss of independence. Globally, the incidence of hip fractures was estimated at 14.2 million cases (95% UI, 11.1–18.1), with an associated 2.9 million years lived with disability (95% UI, 2.0–4.0) in 2019. Falls were identified as the leading cause [5]. The burden of hip fractures is also significantly higher in women than in men [6]. Women demonstrate higher incidence rates (189.7 [95% UI, 144.2–247.2] vs. 166.2 [95% UI, 133.2–205.8] per 100,000) and greater years lived with disability (38.4 [95% UI, 26.9–51.6] vs. 33.7 [95% UI, 23.1–45.5] per 100,000) compared with men. Lower incidence rates have been reported in many low-latitude countries, which may be linked to greater sunlight exposure [5]. Studies conducted across different regions highlight considerable geographic variation, with hip fracture rates generally higher in industrialised countries than in developing regions. The highest incidence is seen in Northern Europe and the United States, while much lower rates are reported in Latin America and Africa [7]. However, the relatively low rates observed in Africa and Latin America may partly reflect underreporting and limited data availability, rather than true differences in incidence. Despite this, a clear global trend is emerging, with developed countries bearing a disproportionately higher burden of neck of femur fractures, largely driven by their increasingly ageing populations [6].

The co-existence of these two conditions is common and gives rise to a complex clinical scenario characterised by substantial thromboembolic and haemorrhagic risks. Atrial fibrillation is most commonly managed with direct oral anticoagulants, which are recommended for men and women with CHA_2_DS_2_-VASc scores of ≥1 and ≥2 respectively. Consequently, a significant proportion of patients with atrial fibrillation who present with a neck of femur fracture are receiving anticoagulation therapy, either with a direct oral anticoagulant or, less frequently, warfarin.

This makes perioperative management more complex, particularly when deciding when to stop and restart anticoagulation. As a result, care often requires input from multiple specialties, including cardiology, haematology, and trauma and orthopaedic teams. One of the main challenges is finding the right balance between thromboembolic risk—which is already increased in patients with atrial fibrillation and further heightened by immobility and surgery—and the risk of bleeding, especially if anticoagulation is resumed too early after surgery or stopped too late beforehand. These challenges will be discussed in detail throughout this article.

## 2. Materials and Methods

A comprehensive literature search was undertaken using PubMed, MEDLINE, Scopus, and Google Scholar to identify relevant studies published between 2000 and 2025. A combination of keywords was used, including “atrial fibrillation (AF),” “neck of femur fractures (NOF),” “anticoagulation,” “orthogeriatric,” “falls,” “bridging therapy,” “perioperative,” “polypharmacy,” “co-morbidities,” and “venous thromboembolism (VTE).” To ensure the search was as thorough as possible, the reference lists of key articles and recent reviews were also manually screened, allowing for the inclusion of additional relevant studies and the grey literature.

### 2.1. Eligibility Criteria

Articles were included if they met the following criteria:

- Focused on the challenges of the interplay of patients with AF when presenting with a NOF in adult populations (≥18 years). The bulk of the articles used focused on the elderly population as that tends to be the population presenting with a NOF.
- Published in US/UK English between 2000 and 2025.
- Presented original data, systematic reviews, narrative reviews, or meta-analyses that addressed epidemiological, clinical, pathological, psychosocial, management and perioperative challenges in people presenting with NOFs who have co-existent AF.

Studies were excluded if they were case reports of singular clinical encounters without broader applicability, non-AF, NOF, anticoagulant-related topics, paediatric populations, or non-peer-reviewed commentaries or editorials.

### 2.2. Study Screening and Selection

Following de-duplication, records were screened in two successive stages. First, titles and abstracts were assessed for relevance according to the eligibility criteria. Articles deemed relevant were then retrieved in full text and assessed independently by two re- viewers. Discrepancies in inclusion decisions were resolved through discussion or in consultation with a third senior reviewer where needed.

### 2.3. Synthesis Approach

Given the diversity of study designs, populations, and outcomes, a narrative synthesis was undertaken. This approach allowed for the integration of both quantitative data, such as prevalence estimates, and qualitative findings, including psychosocial insights. Where possible, results were organised by theme and clinical relevance, and compared with general population benchmarks to highlight differences specific to patients with AF presenting with a NOF. The aim of the synthesis was to uncover patterns, identify gaps, and capture contextual nuances, particularly across different healthcare settings. Although narrative reviews typically do not incorporate formal risk-of-bias scoring, methodological rigour was nonetheless appraised qualitatively. Preference was given to studies with clearly defined diagnostic criteria, transparent methods, and representative sampling.

## 3. Impact of Atrial Fibrillation on Fall Risk and NOF Fracture

The risks of falls leading to significant fractures in the elderly population is multi-factorial. Atrial fibrillation, an irregularly irregular rhythm that can often run fast, can be one of the contributing causes of a fall. AF is considered the prevalent arrhythmia associated with frailty [8]. Older patients who are frailer tend to experience more severe symptoms of AF (EHRA symptom score) [9]. It emerged as a risk factor associated with a high risk of falls [10]. Identification and timely personalised intervention of this risk are crucial in management of falls in elderly (Comprehensive Geriatric Assessment toolkit) [11].

Age is the strongest single risk factor for AF. Multiple age-related mechanisms that promote AF also increase fall risk in older adults. Current evidence clearly explained possible mechanistic links between fall risk and AF in elderly. AF is independently associated with falls and syncope in elderly patients [12]. Pathophysiology, which increases susceptibility of AF, comprises age-related structural and functional changes in atrium. Ageing produces structural remodelling of the atria: progressive fibrosis (often with amyloid), wall stiffening, reduced compliance, and atrial dilation, creating a substrate that favours AF initiation and maintenance. At the same time, electrophysiologic remodelling develops, with slowed atrial conduction, prolonged refractory periods, and reduced sinus node activity (sick sinus, pauses, brady-tachy syndrome) [13]. These changes lead to frequent and persistent AF, bradycardia, or pauses, and rapid irregular tachycardia. In AF, the irregular and often rapid heart rate can lead to inadequate cardiac output, causing transient loss of consciousness or near-fainting episodes [11]. Rapid ventricular rate, characterised by a fast heart rate, can reduce diastolic filling time, leading to decreased cerebral perfusion and syncope.

Both extremes of heart rate and rhythm instability can precipitate pre-syncope/syncope and falls in frail older patients, especially when combined with antihypertensives and rate controlling medications [14].

In addition, AF in older people is tightly intertwined with hypertension, heart failure (especially heart failure with preserved ejection fraction), obesity, diabetes, chronic kidney disease, and other cardiovascular disease. Clinically, these patients have higher lifetime risks of heart failure and stroke, and lose many years free of these complications, reflecting a chronic haemodynamic burden [15]. These haemodynamic changes result in considerable instability, such as orthostatic and exertional hypotension, which in turn significantly increase the risk of falls in the elderly.

Polypharmacy, or the use of multiple medications simultaneously, is common among older adults and carries significant risks, including impaired cerebral perfusion. As people age, they often develop several chronic conditions that require multiple medications, increasing the likelihood of adverse drug interactions and side effects [16]. One key concern is how polypharmacy can affect cerebral perfusion—the blood flow to the brain that is essential for normal cognitive function.

Certain medications, including antihypertensives, anticholinergics, and sedatives, can negatively impact cerebral blood flow. While antihypertensives help control blood pressure, they may sometimes lower it excessively, reducing cerebral perfusion and raising the risk of cognitive decline and falls [17]. Anticholinergics can impair cognition by blocking acetylcholine, a neurotransmitter crucial for memory and learning, with effects seen in both the short and long term [18].

To minimise these risks, healthcare providers should regularly review medications in older adults. Deprescribing—tapering or stopping medications that are no longer necessary—can help improve cerebral perfusion and overall health outcomes [19]. By carefully managing medications, clinicians can reduce the harmful effects of polypharmacy on brain health in the elderly (Table 1).

## 4. Interaction of AF and NOF

Because atrial fibrillation and femoral neck fractures epidemiologically overlap in the elderly, their interaction is largely driven by shared demographics, comorbidities, perioperative and postoperative risks, outcomes, and ultimately morbidity and mortality. A systematic review by Abu-Assi et al. (2019) [20] found that the two conditions frequently coexist and that AF increases readmission and mortality rates among patients with hip fracture. In addition, a population-based examination of 113,600 individuals tracked over 14 years showed a statistically significant increase in odds of incident hip fractures in both men and women diagnosed with AF compared to those without AF, adjusted for factors like age and comorbidities [21]. It concluded that the annual incidence of hip fractures in AF patients was more than double that of those without AF (17.5 vs. 7.4 per 1000 person-years). Combined with results of the systematic review of Abu Assi, it appears that there is a well-characterised clinical presence of AF and hip fractures with established downstream implications, as well as less favourable outcomes on readmission and mortality rates.

Ageing contributes to a rising burden of both AF and hip fractures, a process compounded by increased risk of falls and underlying osteoporosis [4]. Consequently, the heightened fall risk in elderly patients with AF has become a leading cause of hip fracture in this group [12]. A large cohort study demonstrated a strong association between AF and bone fractures, particularly hip fractures [22]. Moreover, complex multimorbidity patterns—including neuropsychiatric disease—in the elderly overlap with AF and elevate fall risk; stratifying these comorbidity patterns can help prevent falls and subsequent fractures [23].

Additionally, elderly patients receiving anticoagulation for primary or secondary thromboembolic prevention experience greater perioperative and postoperative risks. Time to intervention and 30-day mortality are longer in patients with anticoagulation than in those without anticoagulation. The UK HASTE study [24] showed that patients with anticoagulation had longer times to surgery and received fewer interventions within 36 h of admission, leading to higher 30-day mortality. Hospital mortality and postoperative outcomes in older adults with hip fractures are largely predicted by surgical timing; interventions beyond 24 h after admission increase mortality through renal failure, infection, and pneumonia, while early intervention may raise mortality due to acute events such as arrhythmia and electrolyte imbalance, yet delaying surgery does not improve outcomes [25]. Beyond early day morbidity and mortality, AF in the elderly patients after hip surgery is associated with a long-term mortality risk compared with patients without AF. Although the 30-day mortality rate is similar, the 180-day, 1-year, and 3-year mortality rates are significantly higher in the elderly patients with AF [26].

Whilst the majority of the literature focuses on AF as a contributing causative factor to NOFs, it must be acknowledged that a NOF can also result in the development of new-onset AF. This complicates perioperative management. The relationship between these conditions is not one-directional. The development of new-onset AF in the perioperative period following a NOF is significant as it increases one-year all-cause mortality [27] and hence raises the need for closer clinical surveillance for these patients.

The pathophysiology of the development of new-onset AF in the perioperative period is multi-factorial. A review conducted by Bessissow et al. attributes the development of AF to several key factors listed: the activation of the sympathetic system due to the stress of surgery increases heart rate and catecholamines release; temporary perioperative states such as hypovolaemia, intraoperative hypotension, anaemia, trauma, and pain affecting sympathetic activity; disturbances and metabolic imbalances, hypoxia and hypervolemia [28]. Hypoxia can also result in arrhythmia due to pulmonary vein constriction and increase right ventricular pressure and right atrial stretch. Hypoxia can also cause ischaemia of the myocardial atria cells, altering the cardiac electrophysiology conduction pathways. Hypervolemia increases intravascular volume, which causes stretching of the right atrium resulting in AF [28].

Preventing or minimising the factors above which contribute to the development of new-onset AF is important. Equally important is identifying populations at risk. This is crucial in the perioperative periods to avoid poorer outcomes. A South Korean study found that age, COPD, and the echocardiographic parameter of elevated E/e’ ratio were found as significant predictors of postoperative AF in hip fracture surgery patients [29]. Moreover, a Chinese study corroborated the need to risk stratify patients perioperatively by identifying further risk factors to developing a postoperative cardiac event after hip surgery, which include advanced age over 80 years, male sex, a history of arrhythmias, preoperative DVT, and a higher CCI score or ASA classification [30]. By identifying the factors that contribute to new-onset AF as well as highlighting the population most at risk, the aim is to ultimately reduce incidence and improve patient outcomes as AF postoperatively is linked to worse outcomes.

## 5. Perioperative Anticoagulation Challenges and Perioperative Treatment of AF

Balancing surgical timing against perioperative bleeding risk in this patient population presents a significant challenge. In clinical scenarios where timely surgery becomes an important issue of outcome, management becomes especially difficult in patients with atrial fibrillation receiving anticoagulation therapy for primary or secondary prevention of venous thromboembolism. In many of these patients, anticoagulation therapy with direct oral anticoagulants (DOACs), including apixaban and rivaroxaban, or vitamin K antagonists (VKAs), most often warfarin, is prescribed. Hip fracture patients who were on anticoagulation had a longer time to surgery (13.7 h) than controls [31].

To minimise morbidity and mortality, the literature recommends operative management within 36–48 h [32]. Morrissey et al. demonstrated that, although each hour of surgical delay was associated with an incremental increase in mortality risk, this association reached statistical significance when delays exceeded 24 h [33]. Numerous additional studies have corroborated a direct relationship between increased time to surgery and higher mortality and complication rates [34]. Accordingly, surgery should be undertaken as soon as is logistically feasible to optimise patient outcomes.

Should there be a dedicated surgical protocol regarding perioperative management to evaluate the expedited outcome? According to the HASTE study [35], surgical timing was ineffective even with the establishment of dedicated local protocols for perioperative anticoagulation management in femoral fragility hip fracture patients. This occurred across different NHS healthcare systems, which are centrally funded within the UK. Subsequently, the study highlighted variance in local protocol guidance without uniform and standard national guidelines in these patients. This calls for further randomised studies setting standardised protocols to improve care of hip fracture patients with AF on anticoagulation.

Approximately 30–40% of people with hip fractures in the UK are taking anticoagulant/antiplatelet medications pre-operatively. A total of 2–10% of patients are on DOACs, and the majority are on warfarin/vitamin K [36]. These agents confer an increased risk of perioperative bleeding.

When assessing perioperative bleeding risk, two key factors should be taken into account: patient factors, including type of preoperative anticoagulation, thromboembolic disease, renal function, and history of bleeding episodes, and operative factors, such as type of anaesthesia and urgency of surgery.

Accurate preoperative risk prediction using risk tools to reduce mortality and morbidity is crucial in clinical practice while these tools are validated and representative of the population [37]. Over decades, multiple risk stratification tools were used to predict perioperative morbidity and mortality in fragility fracture patients, including Nottingham Hip Fracture score (NHFS), it being the most appropriate tool as per qualitative systemic review [38]. Mostly, they highlighted patients’ frailty and socioeconomic status in predicting 30-day mortality. There is no particular risk tool to assess bleeding risk in hip surgery, which is recognised as a high-risk bleeding procedure. The Canadian Society of Thrombosis implemented a clinical tool for perioperative anticoagulant algorithm comprising interactive information on patients’ type of anticoagulant, age, sex, weight, creatinine clearance, INR, and DOAC level [39].

Nevertheless, the best forward approach to reduce delay and efficient perioperative care in hip fracture patients with anticoagulation is a multidisciplinary approach considering patient factors (comorbidities, renal clearance, bleeding risk associated with surgery, and managing concurrent thromboembolic disease) as well as laboratory assays and the correction of these agents [40].

To optimise preoperative preparedness in patients with anticoagulation, clinical questions regarding when and how to stop and reverse these agents are mandatory. In addition, achieving haemostasis can be improved with agents like perioperative tranexamic acid and blood transfusions when these anticoagulant effects persist in urgent surgery, especially in hip fracture operations.

DOACs typically require a washout period of 24–48 h, depending on renal function and bleeding risk, which may result in unavoidable delays to surgery and adversely affect outcomes. Godier et al. [41]’s multicentre observational study showed that minimal peri-procedural DOAC level (≤30 ng/mL) with negligible anticoagulant effect can be observed in almost all patients with DOAC after 49–72 h discontinuation. This takes longer in patients with moderate renal impairment. Interpretation of safety level to undergo surgery in an emergency has limited data. This should be interpreted in conjunction with renal function and particular DOACs [42].

Although specific reversal agents for DOACs exist (such as idarucizumab for dabigatran and andexanet alfa for factor Xa inhibitors), their use remains limited due to high cost and restricted availability. Consequently, careful risk stratification is essential when considering eligibility for reversal agents, with meticulous assessment of the clinical context.

In the absence of widely available cheap reversal agents, management of DOAC-related bleeding and perioperative care continues to rely primarily on supportive measures and local haemostatic interventions, a practice that is expected to persist until these agents become more accessible and affordable [43]. Again, with haemostatic agents like tranexamic acid, evidence showed that it has reduced perioperative bleeding risk and transfusion rate. However, the national multi-centre audit study (PATHS) [44] in the UK highlighted that the variance of using tranexamic acid in hip fracture surgery among the centres was 0 to 100%. This practice variance could be a lack of long-term effects and safety when using the agent.

While clinical guidelines increasingly favour DOAC use, a small proportion of patients remain well established on warfarin therapy. Warfarin reversal is achieved with vitamin K and, in time-critical situations or cases of major bleeding, is typically administered in conjunction with prothrombin complex concentrates and, less commonly, fresh frozen plasma. In the perioperative setting, taking an accurate medication history, including the timing of the last anticoagulant dose, is crucial. Moving to surgery too soon can increase the risk of bleeding, while unnecessary delays can lead to higher rates of complications. Similarly, after surgery, postponing the restart of oral anticoagulation raises the risk of venous thromboembolism, such as deep vein thrombosis or pulmonary embolism, whereas restarting too early can increase bleeding risk. Striking the right balance requires multidisciplinary collaboration and a patient-centred approach tailored to each individual’s needs.

Whilst anticoagulation medications impose complexities in the perioperative period, other gold-standard medications used in the treatment of AF can also cause complications and require careful considerations and dose adjustments in a perioperative setting of NOFs. AF is typically treated with rate or rhythm control or a combination of both alongside anticoagulation [45]. Those with pre-existing AF may need dose adjustments made to pre-existing medications due to temporary transient perioperative states, such as due to a reduction in renal function or multi-factorial hypotension/hypertension, depending on many variables such as mobility and pain levels. Caution also must be taken in the subset of patients who develop new-onset AF after NOF. Whilst crucial to treat and establish control of rate and rhythm swiftly, the transient perioperative factors mean that initiation of therapy is not as straightforward as non-perioperative settings. There are often many reversible factors in these settings which need addressing as a priority alongside the standard rate/rhythm control [46]. Examples of transient perioperative challenges which contribute to new-onset AF can include electrolyte imbalances and infection. With these reversible causes optimised as much as possible, rhythm control is typically opted for in patients under 65 and rate control is implemented in patients greater than 65 [46]. NICE guidelines state that rate control should be used as the first-line treatment strategy for atrial fibrillation, except in people whose atrial fibrillation has a reversible cause, who have heart failure thought to be primarily caused by atrial fibrillation, with new-onset atrial fibrillation, for those with atrial flutter whose condition is considered suitable for an ablation strategy to restore sinus rhythm, and for whom a rhythm-control strategy would be more suitable based on clinical judgement [47]. This means that the NOF population is typically on a rate control drug for their AF as the vast majority of NOF cases are in the elderly population with pre-existing AF.

## 6. Bridging Therapy

While minimising perioperative bleeding risks, the main practical concern is preventing thromboembolism when anticoagulation is interrupted in patients who need hip surgery. An emerging method to mitigate thromboembolic risk alongside bleeding risk is to utilise bridging therapies. To inform this decision, risk of thrombosis is categorised. If the patient is deemed high risk, then the pathway dictates that they should be bridged.

High-risk thromboembolic patients who should be considered for bridging treatment [42]:

(1) AF patient with previous stroke/TIA in the last 3 months.
(2) Patients with a previous stroke/TIA and three or more of the following: congestive heart failure, hypertension (>149/90 mmH or on medication), age > 75 years, diabetes mellitus.
(3) Patients with a VTE within the previous 3 months.
(4) Very-high-risk patients, such as patients with a previous VTE whilst on therapeutic anticoagulation.
(5) Mechanical heart valve patients, other than those with a bileaflet aortic valve and no other risk factors.

Oxford University Hospital’s guidelines regarding warfarin in the perioperative setting state that it should be stopped 5 days before the operation [48]. The key clinical decision is whether to administer LMWH or, less commonly, unfractionated heparin (UHFH) once the INR drops to less than 2. These local guidelines are based on the wider principle of CHA_2_DS_2_-VASc score to risk stratify individual risk of thrombosis or stroke [49]. CHA_2_DS_2_-VASc score considers several factors: congestive heart failure, hypertension, age, diabetes, prior stroke or TIA, vascular disease, and sex [50]. Scores ≥ 2 generally warrant long-term anticoagulation. INRs are then measured and corrected with vitamin K as necessary. However, Siegal et al.’s meta-analysis [51] concluded that similar thromboembolic risks in perioperative bridged and non-bridged patients increased risk of overall and major bleeding risk in patients treated with vitamin K antagonist. Risks and balance should be outweighed in this cohort.

Whilst clinical trials indicate that most patients taking DOACs for atrial fibrillation do not require bridging anticoagulation, the optimal strategy for patients with a history of VTE is undefined [52]. While warfarin has a long half-life with a variable tail of efficacy after discontinuation, DOACs have a much shorter and more predictable half-life (for example: 12 h for apixaban, 8 h for rivaroxaban) [52,53]; hence, it is futile replacing them with LMWH which has a similar half-life. Therefore, DOACs are widely not bridged in a perioperative setting.

## 7. Thromboembolic Risk in AF Patients Undergoing Hip Fracture Surgery

Multiple studies have demonstrated that temporary interruption of direct oral anticoagulants (DOACs) is safe for most patients with atrial fibrillation (AF), with relatively low rates of major bleeding and thromboembolic events reported. The PAUSE study evaluated patients with AF treated with DOACs using standardised perioperative management strategies that did not involve preoperative bridging. Anticoagulation was withheld on day −1 and the day of surgery for low-risk procedures, and from day −2 before surgery through day 1 postoperatively for high-risk procedures. The study reported low rates of major bleeding (1.35% and 1.85%) and thromboembolic events (0.16% and 0.37%) in the apixaban and rivaroxaban arms, respectively [54]. However, this study did not specifically evaluate patients undergoing hip surgery.

Hip surgery is associated with a substantially increased thromboembolic risk due to marked postoperative reduction in mobility. In addition, patients with AF have a five-fold higher risk of stroke compared with those without AF [55]. These factors necessitate careful, individualised perioperative decision-making, as interruption of anticoagulation increases thromboembolic risk, particularly in this high-risk population. Even in the general population, postoperative thromboprophylaxis is routinely employed, acknowledging the contribution of reduced mobility and the postoperative inflammatory state to increased venous thromboembolism (VTE) risk [56]. In this population, the risk of VTE has been estimated at 4.7%, with most bleeding events consisting of hematomas and few requiring re-operation for wound complications [56]. Importantly, VTE occurred in 1.19% of patients and bleeding events in 3.43% within 30 days postoperatively, in a cohort study of 29,264 patients undergoing total hip or total knee arthroplasty [57]. This study concluded that VTE risk following arthroplasty was primarily driven by underlying patient-specific risk factors. Atrial fibrillation represents one such risk factor, increasing the likelihood of VTE while simultaneously conferring an elevated bleeding risk if therapeutic anticoagulation is resumed prematurely. VTE and bleeding risks also vary according to the type of surgical procedure performed. Hakami et al. demonstrated that, compared with hemiarthroplasty, total hip replacement is associated with an increased risk of VTE, including deep vein thrombosis, without a corresponding increase in pulmonary embolism risk [58]. Consequently, surgical decision-making should be guided by individual patient risk profiles for thrombotic and cardiovascular events.

Furthermore, 9% of patients undergoing total joint arthroplasty have pre-existing AF, and AF in association with total joint arthroplasty, likely lead to higher rates of postoperative complications and, in the case of total hip arthroplasty, longer hospital stays [59]. There are limited studies specifically evaluating VTE risk in patients with AF undergoing hip fracture surgery; therefore, although both conditions independently increase VTE risk, quantifying their combined effect remains challenging. Nonetheless, existing evidence emphasises the importance of careful, patient-centred perioperative planning to address the complexities of anticoagulation management in this patient population.

## 8. Postoperative Anticoagulation Decisions

The timing of restarting anticoagulation postoperatively must balance risk of bleeding from recent surgery and risk of embolic events (particularly strokes). Usual practice is to restart DOACs 48–72 h post-op, provided haemostasis is secure [60]. In the PAUSE trial, it was shown that for low-risk bleeding procedures and high-risk bleeding procedures, DOACs can be resumed 24 h and 48–72 h, respectively [49]. In this case, hip surgery would be categorised as a high-risk bleeding procedure. To determine the thromboembolic risk, three main factors are considered: whether the patient has atrial fibrillation, prosthetic heart valves, or recent thromboembolism (venous or arterial) [61]. CHA2DS2VASc score can be assessed to quantify risk in the AF population. Bleeding risk also needs to be considered.

An equivalent scoring system called HASBLED (Hypertension, Abnormal liver or kidney function, Stroke, Bleeding history or predisposition, Labile International Normalised Ratio [INR], elderly, drugs, alcohol) is also often applied [61]. A score of >3 indicates a high bleeding risk. According to a study conducted by Quintero et al., patients aged ≥ 70 years are at a higher risk of major bleeding after major orthopaedic surgery [62]. This elderly population is generally at higher risk of venous thromboembolism (VTE), as indicated by CHA_2_DS_2_-VASc scores, and often has co-existing atrial fibrillation. Delaying anticoagulation for more than 4–5 days postoperatively may increase the risk of early stroke. In a US study, patients undergoing hip surgery had a 3.9% cumulative probability of ischemic stroke during the first postoperative year [63]. Hip fracture repair and a prior history of stroke were identified as the strongest predictors of this complication [63]. Atrial fibrillation itself is a major risk factor for stroke. Another national study in the US found that postoperative stroke following total hip arthroplasty was associated with older age and undergoing the procedure under general anaesthesia, both in univariate and multivariate analyses [64]. When the frailty index was excluded due to its overlap with functional status, additional factors such as smoking and longer operative duration were also linked to increased risk of postoperative stroke [64]. These are further factors which need to be considered, alongside AF, when deciding when to re-commence anticoagulation.

Furthermore, better postoperative anticoagulation decisions in high-thromboembolic-risk patients and impaired renal function depend on three linked assessments: thromboembolic urgency, procedure bleeding risk, and kidney-function-specific drug handling [65,66]. In renal failure, both thrombosis and bleeding risk are elevated, so standardised protocols often need adjustment rather than the automatic resumption of full-dose therapy [67]. In elective perioperative DOAC management, risk-stratified interruption and resumption without routine heparin bridging produced low 30-day major bleeding and arterial thromboembolism rates; PAUSE resumed DOACs 1 day after low-risk and 2–3 days after high-risk procedures [54].

In renal impairment, dose estimation and timing must follow renal function, because institutional guidelines frequently adjust DOAC holds for kidney function, and using eGFR instead of creatinine clearance in small-body-size orthopaedic patients increased bleeding when dosing was effectively excessive [68]. Renal dysfunction changes both drug clearance and the safety of restart timing. Reviews of CKD/ESRD generally favour UFH when rapid reversibility and titration are needed, while LMWH requires dose adjustment in stages 4–5 CKD and DOAC evidence remain more limited and agent-specific [67] (Table 2).

In bleeding-complicated hip fracture, the literature supports temporarily withholding full anticoagulation, controlling bleeding first, and resuming prophylaxis or chronic anticoagulation only after haemostasis is re-established; exact timing is not standardised and usually depends on drug class, thrombotic indication, wound status, renal function, and anaesthesia constraints [72].

Hip fracture surgery is a high-bleeding-risk setting, and postoperative haematoma can drive wound dehiscence and infection, so management is typically multidisciplinary rather than a protocol-free restart of the preinjury regimen [73]. Bleeding control comes before anticoagulant resumption in this population because preinjury oral anticoagulation is associated with more surgical blood loss and a 1.34-fold higher transfusion risk in meta-analysis, even though postoperative thromboembolism was not clearly higher overall. After bleeding, one reported centre used therapeutic LMWH for at least 1 week and switched back only when wounds were dry and uninfected; this is a reported practice pattern, not a validated standard.

Where neuraxial anaesthesia is relevant, anticoagulants and P2Y12 inhibitors increase spinal or epidural haematoma risk, so restoration of haemostatic competence or use of general anaesthesia matters before restart decisions [74]. Structured protocols appear to help: VKA-reversal pathways shortened time to surgery by 45.3 h without increased bleeding, and orthogeriatric protocols achieved timely surgery without worse postoperative outcomes [75].

For a bleeding-complicated hip fracture, the evidence supports restarting anticoagulation only after bleeding stability and wound haemostasis, with timing individualised by indication and bleeding severity because hip-fracture-specific resumption data remain limited.

Hence, resuming postoperative therapeutic dose of anticoagulation requires careful consideration and multi-disciplinary team discussions as there are many factors which need to be considered when weighing up risk of thromboembolic disease, namely strokes, versus postoperative bleeding risk.

**Figure 1:**
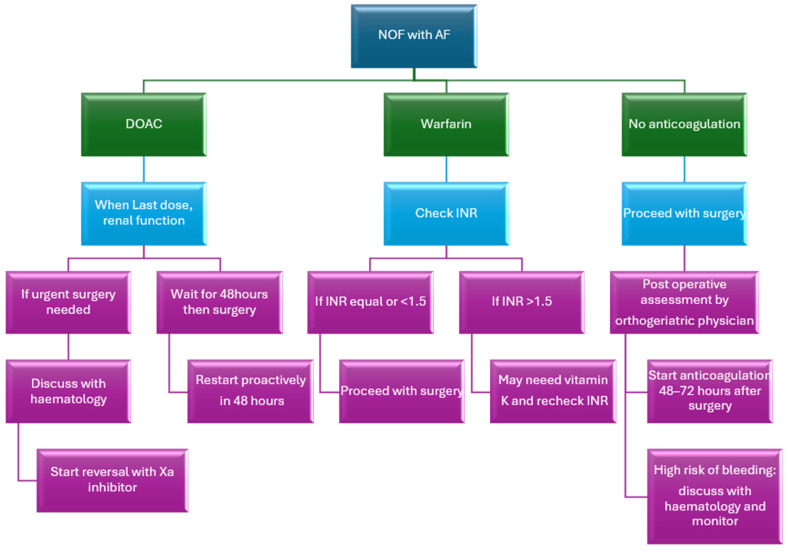
Algorithm for peri and postoperative anticoagulation assessment.

Recent research found improved bleeding profiles in AF patients with the newer factor XI inhibitors compared to DOACs and VKAs, although at the expense of slightly increased or uncertain thromboembolic risk [76]. This presents the possibility of implementing factor XI inhibitors in perioperative settings in NOF treatment due to the improved bleeding profiles. A systematic review and meta-analysis conducted by Toledo Lima de Alcântara et al. earlier this year [77] found that factor XI inhibitors cut major bleeding risk by ∼70% compared with DOACs. Clinically relevant non-major bleeding was reduced by ∼62% with FXI/XIa inhibition [77]. These new findings may revolutionise perioperative anticoagulation issues surrounding NOF surgery. Currently more research is needed to establish their thromboembolic risk, especially in orthopaedic surgical settings such as NOFs. Hence, currently, their use is not widespread in this setting due to this, as NOF surgery increases VTE risk profoundly already, and, with uncertainty around their VTE effects, more studies are needed. Nevertheless, with such excellent results on reduction in bleeding, this class of drugs is definitely one to be aware of for the future in perioperative settings (Figure 1).

## 9. Complications and Outcomes

Patients with atrial fibrillation and femoral fractures undergo a complex clinical course. We have examined the overlap in epidemiology, interactions, treatment challenges, and perioperative and postoperative issues. Finally, we turn to complications and outcomes of patients with AF and hip fracture. Length of stay is indeed longer, and in-hospital complications are frequent among elderly patients with hip fractures. Sepsis, acute kidney injury, respiratory failure, acute arrhythmia, and electrolyte imbalance largely determine in-hospital outcomes and length of stay. Consequently, stays exceeding seven days are strongly linked to complications such as deep vein thrombosis, pulmonary embolism, pneumonia, heart failure, and comorbid conditions [78].

New-onset perioperative atrial fibrillation after hip surgery, occurring in 3.7% of cases, is strongly associated with one-year mortality and prolonged hospital stay [79]. This, in turn, leads to new-onset heart failure during the admission.

Delirium, depression, and dementia are well-known complications after hip surgery in the elderly. Postoperative delirium and neuropsychiatric presentations are common among patients with pre-existing cognitive impairment and mental health issues. Evidence indicates that postoperative delirium predicts longer hospital stays in older adults [80]. A nationwide Danish study found that thromboembolic events such as myocardial infarction and stroke after hip surgery were under-reported. The risk of these events is highest in the postoperative period and then declines over time. However, advanced age, prior cardiovascular risk, and previous events influence the risk [81]. Ultimately, complications after hip surgery in elderly patients with atrial fibrillation affect 30-day mortality and long-term survival, including one-year and three-year mortality. Age, frailty, and higher injury scores are strong predictors of outcomes [78] (Table 3).

## 10. The Orthogeriatric Model

The orthogeriatric model of care refers to a structured, shared-care approach, involving collaboration between orthopaedic surgeons, geriatricians, and the wider multidisciplinary team to co-manage older adults with fragility fractures [82]. Several frameworks of orthogeriatric care have been described in the literature, including routine, consistent geriatric consultation on request in an orthopaedic ward, geriatric-led care with orthopaedic consultation, and the shared-care integrated model where both teams hold shared responsibility [83]. These models may be particularly beneficial in patients with co-existing AF, where orthogeriatric care is key in the perioperative period for the holistic management of the patient’s medical multimorbidity and frailty-associated complications [84]. Therefore, although the advantages of an orthogeriatric model of care have been described in general hip fracture cohorts, there is little direct evidence in patients with co-existing AF. As a result, any suggested benefit in this subgroup should be considered clinically plausible rather than definitively proven. AF patients, a high-risk subgroup, are likely to gain more benefit with an orthogeriatric model able to address both the acute trauma and the structured coordination of multiple comorbidities, than from care designed solely around fracture management, particularly in reduction in mortality and complications [85].

There is consistent evidence from systematic reviews and meta-analyses that orthogeriatric care is associated with reduced mortality compared with standard orthopaedic-led care. For example, there was a 28% reduction in in-hospital mortality and 14% reduction in one-year mortality amongst patients managed within orthogeriatric care models compared to usual care [82]. With regard to the superiority of one model of care compared to another, a meta-analysis demonstrated that any kind of orthogeriatric care model was associated with lower-term mortality (pooled odds ratio 0.85, 95% CI 0.74–0.97), and, specifically, care delivered on a dedicated orthogeriatric ward showed an even greater mortality reduction (odds ratio 0.62, 95% CI 0.48–0.80) [86]. In addition to mortality benefits, orthogeriatric care has been linked to improved acute inpatient outcomes, including reduction in length of hospital stay (mean difference 1.55 days), and reduced incidence of common inpatient complications including delirium, pressure ulcers, and urinary tract infections [82]. While there were methodological differences between the studies, these findings support the overall positive impact of integrated management in hip fracture patients.

As established, anticoagulation decisions are key in AF patients with NOF fractures. Multidisciplinary planning may play a core role in anticoagulation management in the perioperative period through use of scoring frameworks (such as CHA_2_DS_2_-VASc and HAS-BLED or ORBIT scores) and pharmacological agents (such as DOACs). Improved care coordination demonstrated in orthogeriatric care can in turn avoid surgical delay, with one study demonstrating a significant reduction in mean time to surgery from 41.8 to 27.2 h following an orthogeriatric pathway [87]. This is not to suggest that the orthogeriatric model of care replaces or exceeds the expertise of cardiology, anaesthesia, or orthopaedic surgery, but more so that the orthogeriatric model coordinates competing risks, such as bleeding, thromboembolism, and surgical timing, within a shared, integrated pathway.

Importantly, orthogeriatric care appears to enhance both rehabilitation and long-term recovery. Evidence indicates that patients admitted directly to an orthogeriatric ward are more likely to achieve successful rehabilitation compared with those initially admitted to an orthopaedic ward and later transferred for geriatric support [88]. This underscores the value of early geriatric involvement and consistent, continuous care throughout the hospital stay. Furthermore, meta-analytic data suggest a small but statistically significant improvement in health-related quality of life for patients receiving orthogeriatric care compared with standard orthopaedic management [89]. These findings highlight that integrated care models can provide meaningful, patient-centred benefits by supporting rehabilitation and reducing complications over the long term.

Comprehensive geriatric assessments (CGA) involve a structured analysis of an elderly patient’s medical, psychosocial, and functional status [90], which can improve outcomes in hip fracture patients, including improved medication management, reduced polypharmacy, and improved health-related quality of life [91]. This may be particularly relevant in frail AF patients, who often experience worse adverse outcomes including increased risks of stroke, bleeding and all-cause death, and reduced likelihood of receiving oral anticoagulant prescription [92]. The impact of frailty on anticoagulant prescription may reflect concerns regarding bleeding or falls. Orthogeriatric teams can provide specialist input on these complex medical issues [85], particularly weighing up the risks of bleeding, thromboembolism and falls in these patients.

As mentioned, the benefits of the orthogeriatric model are mostly demonstrated from general hip fracture populations, as opposed to populations with concomitant AF. Further research is needed to specifically evaluate orthogeriatric care models in patients with hip fractures and co-existing AF.

## 11. Multidisciplinary Approach

There is a need for a holistic approach in AF with NOF. For instance, alongside addressing the surgical needs of this patient group, the medical comorbidities, analgesia requirements, nutritional deficits, and rehabilitation goals of these patients should also be identified and addressed [93,94]. This forms part of a multidisciplinary team (MDT) approach to patient care, which combines the expertise of a diverse group of healthcare professionals from different fields to best determine a patient’s management plan and improve the quality of their care [95]. This approach is most effective at managing patients with chronic health conditions [96]. MDT collaboration has been found to reduce perioperative thrombotic events in frail orthopaedic patients, likely through development of individualised treatment plans that review medication dosages, bridging therapies and timing of anticoagulation resumption [97], which may be of particular benefit to patients with co-existing AF.

Input from several subspecialties is key in achieving optimal outcomes for patients with NOF fractures and AF. The orthopaedic team is vital in surgically managing the fracture, with timely surgical intervention playing an important role in reducing long-term mortality and perioperative complications [98]. To enable timely surgery, the collaboration of anaesthetists in perioperative planning is key. Anaesthetists assess the risks of proceeding with anaesthesia in frail, elderly and comorbid patients, and establish strategies to minimise anaesthetic-related complications such as hypotension [99]. Given the cardiovascular risks in this patient group, cardiology input allows for timely cardiac stabilisation, including rate and rhythm control, assessment of haemodynamic stability, and balanced discussions regarding anticoagulation in the perioperative period, which is crucial in reducing cardioembolic complications [100]. Input from geriatric care is complementary, rather than hierarchical, in relation to the expertise of orthopaedics, anaesthesia, and cardiology. Geriatric care in fracture patients integrates and coordinates the management of acute medical pathologies, investigation of underlying osteoporosis, conduction of falls assessments, and facilitation of long-term rehabilitation [101].

**Figure 2:**
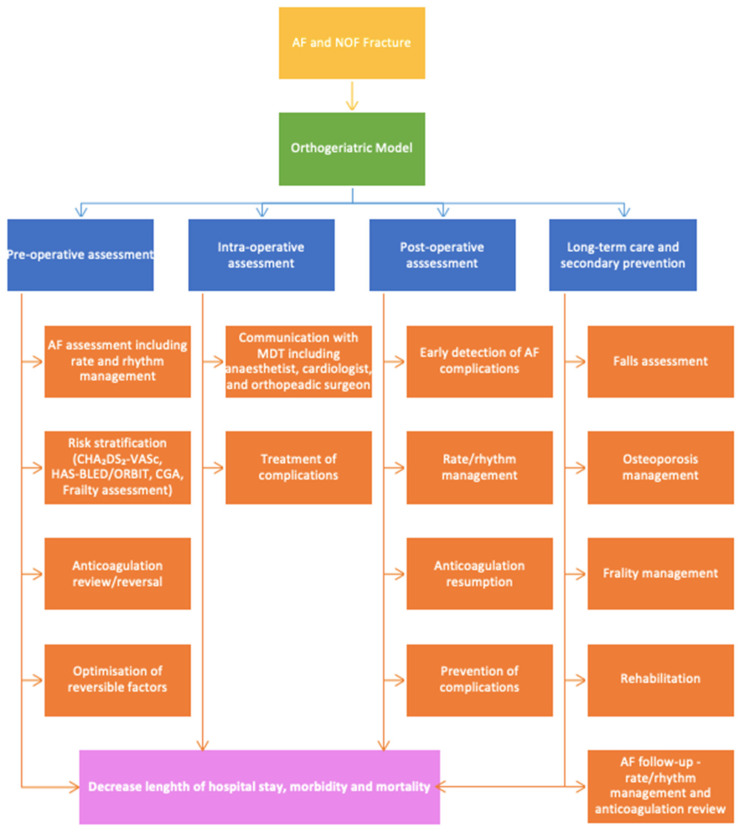
This figure depicts the typical considerations that are embedded in perioperative care in an orthogeriatric setting with the sole outcome of reducing length of stay in hospital as well as reducing morbidity and mortality for the patient population.

It is through these specialist clinicians, and the wider MDT team, that co-ordinated, high-quality care can be delivered to this vulnerable patient group (Figure 2).

## 12. Study Strengths and Limitations

The quality of the evaluated research articles is a significant strength of our assessment. This research was conducted as a narrative review instead of a systematic review and provided current information on the topic. The aim of this narrative review was to provide readers with a thorough evaluation of the issue while simultaneously presenting a succinct update on the subject matter. This review has some limitations. The authors used free search engines for literature retrieval, facing restrictions in fully accessing some articles. The authors included just articles published in English; nonetheless, considering the extensive number of studies in the review, the few excluded studies would have a negligible impact on the study’s results.

## 13. Conclusions

Patients who have both AF and a NOF represent a high-risk group that requires careful coordination between surgical timing, anticoagulation management, and cardiovascular stability. Delaying surgery because of anticoagulation concerns can negatively impact outcomes, while proceeding too early without proper reversal increases the risk of bleeding. Using standardised tools, such as the CHA_2_DS_2_-VASc and HAS-BLED scores, alongside a multidisciplinary care approach, is essential for optimising both short- and long-term outcomes in this vulnerable population.

## Figures and Tables

**Table 1:** Summary of the impact of atrial fibrillation on fall risk and NOF fracture.

	Summary of the Literature	Reference
Age	AF and hip fracture are common in older people. The prevalence of AF increases markedly with advancing age, affecting more than 10% of individuals older than 80 years. AF is considered the prevalent arrhythmia associated with frailty. Similarly, more than 90% of hip fractures occur in individuals over 65 years of age, most commonly as a result of falls and underlying osteoporosis.	[1,4,8,9]
Fall	AF is independently associated with falls and syncope in elderly patients. Falls may lead to a hip fracture and perioperative management can be complicated by AF.	[12]
Comorbidities (diabetes, hypertension, CKD and heart failure)	These comorbidities increase the risk of both AF and the risk of falls that may lead to fractures, especially NOFs. They also require extensive medication so contribute to the issue below of polypharmacy.	[15]
Polypharmacy	Polypharmacy is extremely common in chronic conditions, especially in the elderly patients with AF. It may increase risk of falls and also may increase risk of hip fracture. Antihypertensives particularly contribute to this issue as they reduce cerebral perfusion and can exacerbate postural hypotension.	[16,17,18]

**Table 2:** Renal function factors shaping anticoagulant restart decisions.

Factor	Evidence-Based Implication
Renal function measure	Creatinine clearance is safer than eGFR alone for drug dosing in small-BSA postoperative orthopaedic patients [68]
DOAC restart	Recheck GFR before resumption, especially if postoperative renal decline is possible [69]
Advanced CKD on apixaban	Perioperative management is challenging because clearance is delayed and dialysis has little effect on levels [70]
Regional anaesthesia context	In renal impairment, longer drug-free intervals may be needed because timing should reflect prolonged half-life [71]

**Table 3:** This table highlights how the complex interaction between AF and hip fracture may adversely impact medical and surgical management in older people.

Outcome	Key Evidence from the Literature	Reference
Length of hospital stays	NOF and AF lead to a prolonged length of hospital stay	[41,48,49]
Hospital admission	Systematic review evidence identified AF as a predictor of increased hospital readmission after hip fractures	[20]
Preoperative complications	Surgical delay exceeding 24 h is associated with statistically significant increased mortality risk	[26,27,28]
Intraoperative complications	Patients receiving anticoagulation require complex perioperative management due to increased bleeding risk and anticoagulant reversal	[26,27,28,29]
Postoperative complications	Postoperative VTE risk is 4.7% in surgically treated hip fractures, with most bleeding events being haematomas	[38,41,47,49]
Disability	The cumulative probability of ischaemic stroke in the first year post-hip surgery is approximately 3.9%	[45,47]
Morbidity	AF is associated with increased rates of morbidity during admission for hip fracture	[25,26,27]
Mortality	AF is associated with significantly increased 180-day, -year and 3-year mortality after hip fracture surgery	[5,20,25]
Financial burden	Increased resource utilisation due to prolonged hospitalisation, and DOAC-related bleeding management	[29]

## Data Availability

No new data were created or analysed in this study. Data sharing is not applicable to this article.
